# Supplementary material for: Are carbon emissions trading and green financial instruments synergistic? -Comprehensive quantitative research based on content analysis
Source: PLoS One. 2024 Mar 7;19(3):e0298601. doi: 10.1371/journal.pone.0298601 (PMC10919601; doi:10.1371/journal.pone.0298601)
Supplement: S1 Data — (ZIP) [file pone.0298601.s001.zip › Data/地级市Z_F工作报告.zip!/║■─╧/╥μ╤⌠╩╨/└┤╫╘╣1⁄2╓┌║┼í╛┬φ┐╦╩2╛▌═°í┐_2013.docx]

各位代表：

　　现在，我代表市人民政府向大会作政府工作报告，请予审议，并请市政协委员和列席会议的同志提出意见。

　　一、过去四年的工作

　　四年来，在中共益阳市委的坚强领导下，我们按照“坚持科学发展，奋力后发赶超，建设绿色益阳”的总体思路，团结带领全市人民，扎实工作，开拓进取，实现了经济的平稳较快发展和社会大局的和谐稳定。

　　过去的四年，后发赶超步伐加快，经济实力迈上新台阶。从2008年到2012年，全市生产总值由537亿元增加到1002亿元（2012年的数据为预计数，下同），年均增长13.2%，总量突破千亿大关，在全省排名前进1位；财政总收入由30.08亿元增加到75亿元，年均增长25.7%；固定资产投资由258.4亿元增加到620亿元，年均增长32.9%；社会消费品零售总额由187.9亿元增加到354亿元，年均增长17.5%；年末金融机构本外币各项存款余额由426.1亿元增加到866.8亿元，年均增长15.3%，各项贷款余额由207.7亿元增加到435.7亿元，年均增长16%。

　　过去的四年，产业规模稳步扩大，转型升级出现新变化。从2008年到2012年，规模工业增加值从127.9亿元增加到374亿元，年均增长21.3%，总量排名全省第八，前进4位；规模工业企业由629家增加到815家，产值过亿元企业由82家增加到350家；工业对经济增长的贡献率由42.6%提高到50%；我市连续5年被评为全省推进新型工业化工作先进单位；益阳高新区升格为国家级高新区；汉森制药、太阳鸟游艇、克明面业三家企业成功上市；引进了中联重科等一批重大产业项目；中国驰名商标由1个增加到11个，湖南省著名商标由32个增加到93个，湖南名牌发展到36个，安化黑茶成功纳入国家地理标志保护产品。农林牧渔业总产值由216亿元增加到313.6亿元，年均增长4.9%；农产品加工业增加值由46.6亿元增加到115亿元，年均增长25.3%；市级以上农业产业化龙头企业发展到294家，农民专业合作社发展到755个。第三产业增加值由206亿元增加到355.8亿元，年均增长12.4%；交通运输、商贸流通、餐饮娱乐、房地产业加快发展，电子商务、现代物流、金融保险、文化旅游业稳步提升。转型发展步伐加快，全市高新技术企业发展到79家，高新技术产业增加值年均增长30%，占规模工业增加值的比重达22.6%；三次产业结构比由25.4:36.2:38.4调整为20.7:43.8:35.5，第二产业比重上升7.6个百分点。

　　过去的四年，绿色发展更加注重，两型建设取得新成效。全市万元生产总值和万元规模工业增加值能耗比2008年分别下降18%、45%。化学需氧量、二氧化硫排放强度分别从每万元生产总值8.13公斤、13.25公斤下降为1.44公斤、7.38公斤，我市成为全国全省污染减排工作先进单位。森林覆盖率由53.7%上升到54.4%；创建国家级生态村2个、省级生态乡镇11个；新增国家级湿地公园4个、国家级自然保护区1个、国家级森林公园1个。益阳市“两型”社会建设综合配套改革试验实施方案、益阳东部新区改革建设实施方案等获省政府批准，东部新区招商引资与配套项目建设取得实质性进展。

　　过去的四年，统筹发展扎实推进，城乡面貌有了新改观。四年累计完成农村公路建设4498公里，完成危桥改造251座。投入水利建设资金40.5亿元，完成各类水利工程23.6万个。新建了220千伏益阳东变电站，新建和改造110千伏及以下变电站28座、10千伏以上线路2160公里。完成农村土地综合整治40万亩，新增耕地2万亩，连续多年实现耕地占补平衡。成功创建省级卫生镇1个，市级卫生村29个。新建农村户用沼气池6.1万户。城市创建活动成效显著，获得“中国优秀旅游城市”、“全国双拥模范城”、“国家森林城市”等城市名片。累计完成城镇基础设施建设投资142.2亿元。全市城镇化率达到42.5%。

　　过去的四年，社会建设不断加强，人民生活得到新提升。各项改革成效明显。国有企业改革全面推进，农村配套改革进展顺利；政府信息公开全面推行，政务服务体系进一步完善。财税、金融、教育、卫生、文化、人事、价格等各项改革不断深化。社会事业全面进步。科技创新能力不断提高，目前全市已建立国家级企业技术中心1家、省级工程技术研究中心7家、省级共性技术服务平台3个，有120项成果获省市科技进步奖，我市成为全国科技进步合格市、全国知识产权试点城市；各级各类教育协调发展，完成义务教育化债3.65亿元，义务教育保障水平全面提高，教育质量稳步提升；大力实施文化强市战略，公共文化服务体系逐步健全，文化软实力不断增强；全民医保基本实现，四年累计投入医保资金13.3亿元，参合农民人均门诊费、住院费比医改前分别减少59.5%和41%；全民健身服务体系建设加快；低生育水平持续稳定，我市连续六年被评为全省人口与计划生育工作先进单位。民生民利持续改善。从2008年到2012年，城镇居民人均可支配收入、农民人均纯收入分别由12447元、4527元增加到19893元、7993元，年均分别增长12.7%、15.5%。累计新增城镇就业13.8万人，新增农村劳动力转移就业11万人。五大社会保险参保人数达到520万人次，各项社会保险制度覆盖了所有人群；城乡低保和农村五保全面提标，保障城乡低保对象29.21万人、五保供养4.28万人；住房保障体系不断健全，通过实施保障性安居工程，累计为16.2万户住房困难家庭解决了住房问题。扶贫攻坚与移民后扶工作扎实推进，四年净减少贫困人口6.3万人，直接发放移民后扶资金3.67亿元。减负惠农政策、城乡低收入群体价费优惠政策落实到位。安全生产在较长时间保持良好形势。物价水平得到较好控制。食品药品监督管理得到加强，人民群众饮食用药安全水平不断提高。创新社会管理，强化综治维稳工作责任，社会治安综合治理保持全省先进。

　　各位代表，过去四年的政府工作，面对国际金融危机带来的严重影响，我们直面了困难和压力，付出了艰辛与汗水，收获了成功和喜悦。回顾过去的四年，我们深切体会到：必须坚持绿色发展，把绿色益阳建设作为我市又好又快发展的战略制高点，大力促进资源节约、环境友好，实现全面协调可持续发展；必须坚持工业赶超，突出工业经济的龙头作用，强力推进园区建设，加快优势产业发展，为实现后发赶超奠定强有力的产业基础；必须坚持投资拉动，把项目建设作为加快经济社会发展的强力支撑，通过项目建设拉动投资增长，夯实发展基础，增强发展后劲；必须坚持改革创新，挖掘加快发展的内在动力，努力破解发展中的矛盾和问题，不断增强发展活力；必须坚持以人为本，扎扎实实为人民群众办实事，大力保障和改善民生，努力维护社会和谐稳定，营造良好的发展环境。

　　二、2012年工作回顾

　　2012年，是我市近年来经济发展最困难的一年。一年来，全市上下突出发展第一要务，团结奋进，保持了经济社会的平稳较快发展。全市生产总值比上年增长12%，财政总收入同口径增长25%，固定资产投资增长35%，社会消费品零售总额增长15.3%，城镇居民人均可支配收入和农民人均纯收入分别增长14.8%和18%，年末金融机构本外币各项存款、贷款余额比年初分别增加137.8亿元、75.9亿元。

　　（一）突出稳中求进，产业建设取得成效

　　农业经济稳步发展。农林牧渔业总产值比上年增长4.4%。农产品加工业增加值比上年增长26%。农村土地流转面积达269万亩，其中土地信托流转面积26.5万亩。推进水利建设“十大工程”，投入水利建设资金16亿元，比上年增长33.3%；有效抗御了八轮强降雨和湖区历时20天的超警洪水，防汛抗灾工作取得胜利。

　　工业经济较快增长。全市工业总产值突破1400亿元，比上年增长18.5%；实现工业增加值393亿元，比上年增长14.1%，其中规模工业增加值增长15.5%。新进规模工业企业73家。装备制造（含船舶工业）、食品加工、电子信息三大优势产业的产值，占全部规模工业企业总产值的一半，主导和带动作用突出。

　　第三产业平稳发展。全市第三产业增加值比上年增长12.5%。交通运输、商贸流通、房地产、旅游业等稳步增长。

　　（二）突出两大会战，项目建设稳步推进

　　交通建设大会战扎实开展。完成交通建设投资70亿元。石长铁路复线建设稳步推进；二广高速、杭瑞高速益阳段正在抓紧建设，319国道益阳南线高速全线开工；常安高速桃马连接线、益阳新港进港公路建成通车，益桃一级公路提质工程顺利竣工；南茅运河航运工程、沅江黄茅洲大桥、沙头资江大桥等超额完成年度投资任务。完成农村公路建设600公里、危桥改造232座。

　　园区建设大会战强力实施。九大工业园区完成投资163亿元，建成标准化厂房60万平方米；签约落户项目156个，新开工项目170个，新投产项目140个；实现工业增加值200亿元，其中规模工业增加值180.5亿元，比上年增长16.1%；实现税收13.9亿元，增长27.7%。

　　重点工程“三项行动”取得实效。74个“三项行动”项目完成投资131亿元。

　　（三）突出城市创建，城镇功能不断完善

　　全国文明城市、国家卫生城市、国家交通管理模范城市、省级创业型城市等创建工作全面推进。

　　规划编制与执法得到加强。《益阳市城市总体规划》修编工作进展顺利，编制了《益阳市城乡统筹专项规划》、《城市综合交通体系规划》等专项规划。扎实开展中心城区违法建筑和非法房地产开发专项整治，中心城区违法建筑蔓延态势得到有效遏制。

　　城市建设与管理水平提高。中心城区完成市政基础设施建设投资24.9亿元，完成了迎宾路等主次干道和293条背街小巷的提质改造，完成了第四个“十万株树进城”任务。大力开展十大专项整治行动，全面落实“门前三包五不准”管理办法，市容市貌全面改观。31个农贸市场的新建和提质改造、14个城中村及8个城乡结合部的整治达标工作基本完成。

　　小城镇建设步伐加快。完成城镇基础设施建设投资51.47亿元。城镇规划、建设和管理水平提高，综合服务和辐射带动能力增强。马迹塘、梅城、茅草街等交通枢纽重点镇发展迅速。

　　（四）突出改革开放，发展活力日益增强

　　各项改革深入推进。统筹城乡发展改革创新取得突破，探索了符合益阳实际的“三集中”、“六一体”、“四基础”统筹城乡发展新模式。行政审批制度改革、市直国有企业改革、文化管理体制改革、集体林权制度改革、水务管理体制改革等都迈出新的步伐。

　　立项争资和招商引资成效明显。全市争取上级项目资金130亿元，比上年增长16%。实际利用市域外资金310亿元，完成固定资产投资226亿元，比上年增长21.6%。其中，直接利用外资1.42亿美元，增长15.8%；利用境内省外资金165亿元，增长21.4%。益阳成为“湖南省服务外包示范基地”。

　　资本市场建设取得新的突破。克明面业在深交所上市。24家企业纳入省级重点上市企业后备资源库。新增6家小额贷款公司和5家融资性担保公司。益阳市农村商业银行组建进入申请筹建阶段。华融湘江银行益阳分行、沅江浦发村镇银行开业。市城建投成功发行16亿元企业债券。

　　（五）突出统筹协调，发展方式逐步转变

　　统筹城乡发展取得成效。农村土地信托流转经验在全省推广，“草尾模式”在全市26个乡镇推开。农村集体土地和房屋确权颁证工作稳妥实施。启动和建设了17个农民集中居住区。330个村完成了“四位一体”机制建设。城乡统筹规划、农村清洁工程、农村土地综合整治等加快推进。

　　节能减排成效明显。全市万元生产总值能耗比上年下降3.95%，万元规模工业增加值能耗下降18%。实施减排项目63个。二氧化硫、氮氧化物、化学需氧量、氨氮排放比上年分别削减5%、10.5%、1.5%和2%。

　　环境保护得到加强。开展了危险化学品、重金属、危险废物专项检查，加强了农村环境综合整治、矿山地质环境治理和中心城区沟渠治理，实施了省里部署的“十大环保工程”，市域环境质量持续改善。

　　科技创新的支撑与引领能力不断提升。新增省级工程技术研究中心等省级创新平台3家，新增高新技术企业25家。8项科技成果获省科技进步奖。年专利申请量突破1400件。省级创新型企业数量排名全省第三。高新技术产业增加值84.4亿元，比上年增长32%。

　　（六）突出社会建设，民生得到有效改善

　　社会事业全面发展。各级各类教育协调发展，“普九”成果进一步巩固，建成义务教育合格学校83所、农村公办幼儿园10所，中心城区义务教育学校“两扩两建”工程顺利实施，校车安全管理工作得到加强，实现了中小学、幼儿园校车服务覆盖50%的省定目标。公共文化服务体系基本建立，完成了益阳历史文物陈列馆建设，完善了48个乡镇综合文化站和17个社区文化活动站（室）的建设，新建了20个周末广场电影放映点，建成农家书屋343家，解决了近5.1万户广播电视盲村用户的收视问题。医药卫生体制改革不断深化，医疗卫生服务体系逐步健全，疾病防控能力进一步增强，卫生应急水平不断提高，医患纠纷比上年下降50%。中心城区“八小”行业专项整治成效明显，食品药品安全监管得到加强。国家免费孕前优生健康检查全面推广，优质服务水平稳步提升，人口计生工作保持全省先进。全民健身运动深入开展，竞技体育成绩突出，益阳籍运动员田卿夺得伦敦奥运会羽毛球女子双打冠军。人才队伍建设得到加强。工商行政管理、质量技术监督工作取得成效。国土资源管理取得成绩，重点项目建设的用地需求基本得到保障。国防动员和后备力量建设扎实推进，国防教育不断加强。广播电视、报刊网络、新闻出版、统计、审计、档案、住房公积金、民族、宗教、外事、侨务、老龄、对台、科协、地方志、消防、气象、水文、供销合作、扶贫、移民、爱国卫生、残疾人、红十字会等各项工作取得新的成绩，工会、妇联、共青团等群团组织在经济社会发展中发挥了积极作用。

　　人民群众得到更多实惠。省政府下达的18项实事22个指标全部完成。新增城镇就业3.31万人，新增农村劳动力转移就业4.75万人，零就业家庭动态就业援助率100%。五大社会保险新增参保人数34万人，征收各类社会保险费22亿元；城乡居民养老保险制度实现了全覆盖。全面完成了保障性住房建设任务。完成了14个乡镇邮政局（所）的补建和240个村电网新建改造。改扩建乡镇敬老院23所。城乡低保和五保供养标准进一步提高，社会救助和保障标准与物价上涨挂钩的联动机制全面实施。医疗救助覆盖城乡，实施社会医疗救助22.7万人次。法律援助有效实施。五个区县（市）第一轮对口帮扶安化贫困乡镇工作圆满结束，“两项制度”衔接直接帮扶贫困农民19500人。移民培训和产业开发取得成效，特困移民避险搬迁安置3168人。

　　（七）突出和谐发展，社会大局保持稳定

　　民主法治建设得到加强。“六五”普法工作得到落实，法治益阳建设全面启动，“法律六进”法制宣传教育活动深入开展，“法治城市（县、市、区）”等创建活动不断深化。村务公开民主管理工作得到加强，第八届社区换届选举工作顺利完成。

　　精神文明建设深入推进。“大爱益阳”第二届道德模范评选表彰活动成功举办。文明村镇、文明单位及其他群众性文明创建活动深入开展。

　　安全生产形势基本稳定。全面开展“打非治违”专项行动，深入排查治理安全隐患，共关闭非法生产经营单位480多家，排查治理各类安全隐患近2万处，督促企业投入安全生产整改、技改资金1.4亿元。

　　综治维稳工作成效显著。社会管理创新重点项目建设取得突破。强化热点整治，保持严打态势，突出治安防范，社会治安形势保持稳定。坚持用群众工作统揽信访工作，信访渠道进一步畅通，赴省进京非正常上访批次明显减少。加强矛盾纠纷排查化解，建立健全“三调联动”机制，一系列不稳定因素得到及时化解与处置。

　　（八）突出职能转变，自身建设得到加强

　　“民本政府”建设取得实效。以市长热线、信访接待等为主通道，主动对接老百姓诉求，解决了一批事关人民群众切身利益的实际问题。“效能政府”建设稳步推进。全面推进政务公开，加快推进行政审批规范化电子化，转变机关工作作风，行政效能进一步提升，发展环境不断优化。严格控制机构编制，行政成本进一步降低。“法治政府”建设得到加强。主动接受人大及其常委会的法律监督、工作监督，自觉接受人民政协的民主监督，认真办理人大代表建议和政协委员提案；大力推进依法行政，行政程序法治化建设等工作取得新进展。“廉洁政府”建设不断深化。强化廉政教育，注重围绕规范权力运行创新体制机制，深入开展纠风治乱，不断健全行政问责制，严查腐败案件，廉洁政府建设取得了新的成效。

　　各位代表，成绩来之不易，经验弥足珍贵。各项成绩的取得，是中共益阳市委正确领导的结果，是市人大、市政协监督和支持的结果，是全市人民团结拼搏的结果。在此，我代表市人民政府，向辛勤工作在各行各业的全市广大干部群众，向各位人大代表、政协委员，向离退休老同志，向各民主党派、工商联、无党派人士和各人民团体，向中央和省属驻益单位，向驻益人民解放军、武警官兵和政法公安干警，向所有关心、支持和参与益阳改革发展的各界朋友，表示衷心的感谢！

　　在肯定成绩的同时，我们也清醒地认识到当前我市经济社会发展面临的困难和问题。主要是：经济总量不大，特别是人均水平不高，转方式、调结构的任务很重；部分重大项目建设进程趋缓；财政收支矛盾依然突出；改善民生任务艰巨，解决就业、就学、就医、出行和食品安全等问题迫切需要有新举措、新成效；维护社会稳定压力较大；政府部门的服务效能需要进一步提升，等等。对这些问题，我们将高度重视，在今后的工作中认真加以解决。

　　三、未来五年发展的总体要求和主要任务

　　未来五年，是益阳后发赶超的关键时期，是益阳全面建成小康社会的攻坚时期，是益阳机遇与挑战并存的战略机遇期。从总体上看，我市加快发展面临诸多有利因素。国际经济发展向好的趋势没有改变，我国发展仍处于可以大有作为的重要时期，经济发展方式加速转变，沿海产业加快向中西部转移，中部崛起面临新的机遇；湖南“3+5”城市群建设提速，洞庭湖生态经济区规划建设加快推进，益阳中心城区纳入长株潭都市区，益阳东部新区纳入“两型社会”建设示范区，安化纳入武陵山片区区域发展与扶贫攻坚规划试点县，益阳环省会比较优势和后发优势逐步显现，将为我们对接和融入长株潭、跨越式发展提供更多的战略契机。因此，我们一定要正确研判形势，坚定发展信心，抢抓发展机遇，提升发展质量，努力开创科学发展、后发赶超新局面。

　　未来五年工作的总体思路是：以邓小平理论、“三个代表”重要思想、科学发展观为指导，全面贯彻党的十八大精神，按照省委推进“四化两型”建设和“两个加快”、“两个率先”的战略部署，紧紧围绕“坚持科学发展、奋力后发赶超、建设绿色益阳”的总体思路，扎实推进交通建设和园区建设两大会战，坚持把新型工业化作为后发赶超的第一推动力、把招商引资作为富民强市的第一选择、把人民群众的满意度作为检验发展成效的第一标准，全面实施工业强市、绿色发展、开放带动和城乡统筹发展战略，为建设经济繁荣、社会进步、生态优美、人民幸福的美丽益阳而努力奋斗。

　　未来五年的奋斗目标是：生产总值年均增长13%，到2017年人均生产总值达到4.6万元，财政总收入年均增长20%，固定资产投资年均增长25%，社会消费品零售总额年均增长17%，规模工业增加值年均增长18%，居民人均可支配收入年均增长12%以上，万元生产总值能耗年均下降3.5%，城镇登记失业率控制在4.5%以内，人口自然增长率控制在7‰以内。

　　实现上述目标，必须加快推进“五个益阳”建设：

　　一是深入建设绿色益阳。建设绿色益阳，转变发展方式，是加强生态文明建设、推动全面协调可持续发展的重大战略举措。要发展绿色生产，大力发展绿色工业、生态农业和现代服务业，抓好节能减排，发展循环经济。要倡导绿色消费，增强全民节能意识、环保意识、生态意识，形成合理消费的社会风尚。要建设绿色生态，加强环境保护，推进林业生态工程和城乡清洁工程，促进生态环境明显好转。要培育绿色文明，建设绿色街道、绿色企业、绿色学校、绿色机关、绿色社区和绿色园区，实现绿色发展、循环发展、低碳发展。通过五年努力，全市绿色发展战略全面落实，绿色文明建设取得新的成效，人居环境得到显著改善。

　　二是加快建设财富益阳。建设财富益阳，提升全市财富总量和人均财富值，是实现富民强市的根本要求。要大力发展实体经济，进一步掀起全民创业热潮，让一切创造财富的活力竞相迸发。千方百计增加居民收入，激活各种生产要素，让一切创造财富的源泉充分涌流；强力推进项目建设，加快培育新的财富增长点，促进财政收入持续快速增长；引导企业加强技术创新，实施科学管理，提高生产经营效益。通过五年努力，全市财富创造能力显著增强，财政综合实力和人民富裕程度显著提升，人均生产总值和居民人均可支配收入达到全省平均水平。

　　三是奋力建设民本益阳。建设民本益阳，着力保障和改善民生，是政府一切工作的出发点和落脚点。要不断加大民生投入，着力解决影响民生的突出问题；千方百计促进就业，巩固民生之本；加快发展各项社会事业，全面建成覆盖城乡的社会保障体系，推进基本公共服务均等化，在学有所教、劳有所得、病有所医、老有所养、住有所居上持续取得新进展，不断增进人民福祉。到2017年，全市民生状况得到较大改善，人民物质文化生活水平全面提升，人民群众的幸福指数大幅提高。

　　四是全面建设法治益阳。建设法治益阳，是维护人民利益、构建和谐社会的客观需要。要加强法制宣传教育，增强全社会法治意识；加强法治政府建设，不断提升政府服务水平；加强对权力运行的制约与监督，提高政府和部门依法行政的能力和水平；完善基层民主制度，保障人民群众的民主权利；加强和规范法律服务，维护社会公平正义。通过五年努力，使法治益阳深入人心，公共权力运行规范，公民合法权益得到切实保障，全社会法律素质普遍提高，法治在社会管理中的作用得到进一步发挥。

　　五是着力建设文化益阳。建设文化益阳，是提升发展软实力，实现在更高层次上又好又快发展的重要抓手。要深入挖掘独具特色的益阳文化，大力实施“六个一”文化工程，扎实开展群众性精神文明创建活动，健全公共文化服务体系，加快文化产业发展，繁荣文艺精品创作生产，加强文化人才队伍建设，进一步解放和发展文化生产力，不断丰富人民精神文化生活。到2017年，社会主义核心价值观深入人心，人民群众精神文化生活更加健康丰富，文化软实力全面增强，文化对经济发展的支撑作用更加显现。

　　四、2013年的发展目标和工作重点

　　2013年是党的十八大召开后的第一年，也是新一届政府的届首之年，开好局、起好步意义十分重大。2013年我市经济社会发展的主要预期目标是：生产总值增长12%，人均生产总值达到2.59万元，财政总收入增长20%，固定资产投资增长25%以上，规模工业增加值增长18%，社会消费品零售总额增长16%，居民人均可支配收入增长13%，城镇登记失业率控制在4.5%以内，万元生产总值能耗下降3.5%，化学需氧量、二氧化硫、氨氮、氮氧化物排放量控制在省定计划之内，人口自然增长率控制在7.41‰以内。

　　重点是抓好十个方面的工作：

　　（一）强力推进两大会战，加快新型工业化进程

　　强力推进交通建设大会战。全年完成交通建设投资100亿元以上。确保沙头资江大桥、沅江黄茅洲大桥竣工通车，确保杭瑞高速益阳段、S308柘马段、S205桃江段建成投入使用；确保益娄、马安、益马、益南高速全面开工；加快推进石长铁路复线益阳段、二广高速益阳段、G319益阳南线高速、S225安化段、S206灰山港至宁益桥、资北干线（资阳—安化）等项目建设；完成农村公路建设500公里。同时，切实抓好其他基础设施及产业发展、民生工程等重大项目建设，协调解决融资、用地等问题，稳妥推进征地拆迁，优化项目建设环境，确保项目早开工、早建成、早投产。

　　强力推进园区建设大会战。建成标准化厂房100万平方米。突出抓好水、电、路、气等基础设施建设。严格落实园区管理考核制度，尽快理顺完善国家级和省级园区的管理体制，把园区建成体制创新的平台、招商引资的载体、产业转移的基地和两型建设的窗口。加快益阳高新区东部产业园、益阳东部新区建设，真正发挥新的增长极的重要作用。

　　加快优势产业发展。围绕装备制造、食品加工、电子信息等重点产业，积极吸纳关联度高、配套性强的企业落户，推动企业集群发展。鼓励优势企业加强技术创新，形成集研发、生产、配套服务和流通于一体的完整产业链，推动产业优化升级。大力培育发展新材料、新能源、文化创意等战略性新兴产业，构建多极发展、多点支撑的产业格局。

　　积极扶持企业发展。落实促进中小微企业发展的各项政策，加大对企业发展的帮扶力度。继续搭建政银企担合作平台，切实缓解企业融资难问题。加强生产要素调度，解决企业发展中的用地用工等问题。

　　（二）强力推进城乡一体化，大力发展现代农业

　　大力推进城乡统筹发展。坚持改革推动，完善城乡一体化体制机制，抓好统筹城乡发展各项工作，着力在城乡规划、基础设施、公共服务等方面推进一体化。实现全市城乡规划工作全覆盖。进一步巩固和扩大农村土地信托流转工作成果。全面完成农村集体土地所有权确权颁证各项任务，其他“三证一图”完成一个乡镇的试点任务。每个区县（市）建设或建成的农民集中居住区达到2个以上。完成全市50%以上的行政村“四位一体”机制建设任务。进一步抓好农村清洁工程，全市所有行政村都达到“四有两无清洁村”标准。整合涉农资金，加大统筹城乡发展投入力度。扎实抓好农村金融综合改革试点工作。全面落实各项强农惠农富农政策。

　　着力提升现代农业发展水平。以农业增效、农民增收为核心，大力发展现代农业。加快发展粮棉油、蔬菜、黑茶、中药材等产业。扎实推进畜禽标准化规模养殖和水产健康养殖。大力发展农产品精深加工。推进农业标准化生产，加快“三品一标”产品发展。加大农业科技创新和推广力度。全力推进农产品物流中心建设。进一步提高农业机械化水平。大力发展高效生态林业。

　　改善农村基础设施条件。加强农村公路建设，抓好危桥、渡口等附属设施改造。继续大兴水利，加快水利“十大工程”建设，抓好一线防洪大堤和内湖渍堤的培修加固，强化堤防管理，加强大中型灌排泵站的更新改造，加快小型农田水利工程建设，继续搞好灌区续建配套改造。完成皇家湖电排建设，启动兰溪哑河、志溪河整治开发前期工作。兴建一批跨村连片的规模供水工程，完成一批病险水库的除险加固，完成上型号水库的实时监控系统建设。继续推进农村电网升级改造工程。加强农村通信基础设施建设。

　　加大扶贫和移民工作力度。加大对贫困地区的扶持力度，加快贫困人口脱贫步伐。坚持“开发性移民”方针，加强库区和移民安置区基础设施建设、产业开发和智力移民，继续抓好特困移民避险搬迁安置，确保库区和移民安置区和谐稳定。

　　（三）强力推进现代服务业发展，努力扩大消费需求

　　大力发展生态旅游业。完成全市旅游总体规划修编，完善旅游产业发展战略。以中心城区和东部新区为核心，打造以一小时车程为半径的休闲度假旅游圈；以安化茶马古道为龙头，打造黑茶文化旅游精品线；以沅江五湖、大通湖内湖为重点，开发南洞庭湿地生态旅游带。完善桃花江竹海、皇家湖旅游度假区配套设施。抓好德昌公园、厂窖惨案纪念馆等红色旅游景区建设。加强星级旅行社、星级饭店、等级景区创建工作，抓好旅游市场促销和旅游人才培训工作。培植一批旅游商品生产、销售示范企业。

　　加快发展现代服务业。以益阳服务外包产业园建设为重点，大力发展呼叫中心、动漫制作、软件开发外包、研发设计外包、数据处理、财务核算等服务外包，支持惠普集团、金蝶软件、搜空高科、恒翔动漫、青苹果数据中心等重点企业发展。进一步加强政策引导与扶持，大力发展科技研发、节能环保、融资租赁等各类新兴服务业态，着力引进一批带动作用明显的现代金融、现代物流和文化创意企业。

　　改造提升传统服务业。大力发展连锁经营、物流配送、代理制等现代流通方式，加快推进数字认证、电子支付和电子数据交换等先进管理模式在传统服务业中的应用。支持商业老字号和服务名牌企业的发展。全面提升餐饮、住宿、家政等传统服务业的发展水平。加强特种行业管理，确保拍卖、典当、报废汽车回收等特种服务行业健康有序发展。

　　积极拓展城乡消费。加快建立扩大消费需求的长效机制，释放居民消费潜力。支持城市现代商业网络向农村延伸，构建城乡一体化的营销网络，畅通农产品进城和工业品下乡的双向流通渠道。加强农村现代市场体系建设，积极发展农超对接、平价商店等多种产销衔接方式，充分发挥供销社在农村现代流通中的主导作用。加快顺德城—益阳家居博览中心、兰溪粮食产业园、农产品批发交易市场、竹制品市场等专业市场建设。大力培育汽车、住房、教育、旅游、健身等新的消费热点。促进房地产业健康发展。

　　（四）强力推进新型城镇化，切实加快城镇发展步伐

　　提高城乡规划和管理水平。充分发挥规划的引领和调控作用，抓好中心城区总体规划修改和报批，完成控制性详细规划的编制和审批，加快编制提升城市景观品位的专项规划，两年内完成修建性详规的编制。完成县城和重点镇控制性详规和修建性详规的编制。加强规划执法，强化中心城区“属地管理、以区为主、市区联动”的控违拆违长效机制，进一步加大控违拆违工作力度。

　　大力加强城镇建设。加强城市经营，加大城建投入，实施一批城市重大建设项目，以大投入促大建设大发展。完善基础设施配套建设，突出加强道路、广场的提质改造，抓好资江两岸、益阳大道、迎宾路、银城南路等城市景观改造工程和益阳石码头历史街区的保护与修缮工程。抓好县城的扩容提质，将县城建设成为县域经济的核心带动极。加大小城镇基础设施建设力度，增强小城镇产业发展、公共服务、吸纳就业、人口集聚功能。

　　切实抓好城市创建和城镇管理。以城市创建活动为抓手，提高城镇管理水平。全面推进全国文明城市、国家卫生城市、国家交通管理模范城市、法治城市等创建活动，确保实现创建国家卫生城市的省预检、国检达标。加强“数字城管”建设，健全长效管理机制，深入推进专项整治，着力改善市容市貌，提升城市品位。加快桃江县、沅江市、安化县创建省级卫生县城步伐。鼓励有条件的小城镇创建省级卫生镇。引导小城镇加强管理，实现镇容秩序的明显好转。加强社区建设，发挥社区在城镇管理中的重要作用。

　　（五）强力推进生态文明建设，全面提升绿色发展水平

　　启动生态城市创建工作。切实加强生态建设和环境保护。大力植树造林，保护好生态公益林、自然保护区、省级以上重要湿地、森林公园。加强水环境、居住环境治理和保护。加强矿山整治。全力实施全省十大环保工程项目，进一步强化环保执法，着力解决突出环境问题。深入开展环保专项行动，积极推进城乡环境综合整治试点示范。加快推进洞庭湖生态经济区规划建设。

　　加快两型社会示范建设。统筹推进绿色益阳与两型社会建设。加强目标引领，抓好两型理念的宣传教育和两型技术的推广应用，深入开展两型示范创建活动。启动益阳东部新区区域道路和鱼形山水库补水工程建设，加快“江南古城”一期工程建设。同步推进两型示范区建设十项改革。

　　大力抓好节能减排工作。严格控制新上高耗能、高污染项目，加快淘汰落后产能。深入开展企业节能行动，大力发展循环经济。突出抓好工业企业污染治理、生活污染治理、规模化畜禽养殖场污染治理、机动车氮氧化物减排专项行动，加快重点建制镇污水处理厂建设。推广节能减排新技术，实施节能减排重点工程，发挥好重点项目的节能减排效益。

　　（六）强力推进改革开放，不断注入发展动力

　　全面深化各项改革。深入推进行政审批制度改革，进一步简政放权、优化程序。基本完成市属国有企业改革。加强和完善机构编制管理。着力推进人事制度改革。大力推进税收协控联管，进一步完善市区、县乡财政管理体制，积极推动国库集中支付改革扩面，全面推行公务卡改革，扎实有序推进预算绩效管理工作。进一步推进医药卫生体制改革。稳步推动水务管理体制改革。继续深化集体林权制度改革。

　　全力扩大招商引资。着力提高招商引资质量。突出抓好工业招商，特别是抓好优势产业和战略性新兴产业招商，做大工业经济总量。突出抓好园区招商，引导园区引进知名企业和重大项目，打造具备主导产业的特色园区。突出承接产业转移，重点承接绿色环保、附加值高、发展潜力大的产业。突出抓好集群招商，加强与各类协会、商会的招商对接，注重引进行业地位高、市场前景好、带动能力强的核心产业集群。实现内联引资形成固定资产投资260亿元以上，实际利用外资1.6亿美元以上。大力发展进出口贸易，进一步提高对外开放水平。

　　积极抓好立项争资。围绕国（省）投资重点，精心谋划一批重大产业项目、民生项目和基础设施项目，积极向上对接，争取更多的项目资金支持。力争全年到位资金150亿元以上。

　　（七）强力推进要素市场建设，切实增强发展活力

　　注重发挥科技引领支撑作用。全力推进产学研政金结合，组建一批新的产业技术创新战略联盟。进一步扩大与高等院校、科研院所的合作。加快企业技术创新平台建设，提高企业创新能力。加强科技成果转化服务载体建设。加大知识产权保护工作力度。加快信息化步伐，大力推进“数字益阳”建设。

　　大力推进融资体系建设。引导金融机构加大信贷投放力度，力争新增贷款100亿元。做大做强做优市级投融资平台。大力推进小额贷款公司试点工作。加快推进融资担保体系建设。实现益阳市农村商业银行挂牌开业。继续引进市域外商业银行到益阳设立分支机构。积极推动未设立村镇银行的区县设立村镇银行。全力推进企业上市工作。积极发展债券市场。引导推动保险行业做大做强，进一步扩大农村政策保险保障范围和覆盖面。大力开展社会信用体系建设，严厉打击非法集资和恶意逃废金融债务行为，不断优化金融生态环境。

　　集约节约利用土地资源。充分挖掘、盘活存量土地。加快用地报批，扩大用地规模，努力保障工业园区、保障性住房、交通建设、民生工程等重点项目用地。严守保有耕地410.4万亩的保护“红线”。抓好环洞庭湖基本农田建设重大工程。

　　加强人才队伍建设。切实抓好行政管理人才、企业经营人才、文化专业人才等人才队伍建设。围绕产业发展重点领域和重点环节，组建一批科技创新团队，建设人才高地。

　　全力优化经济发展环境。妥善处理依法行政、强化监管与支持发展、优化服务的关系，努力在改善发展环境方面实现突破。加强政务中心和公共资源交易平台建设，规范行政权力，优化政务服务。围绕重大项目和重点企业，大力整治施工建设环境和企业发展环境。严厉查处影响发展环境的人和事。

　　（八）强力推进民生改善，稳步提升人民群众幸福指数

　　着力抓好就业再就业工作。大力推进省级创业型城市创建工作。做好以高校毕业生为重点的青年就业工作和农村转移劳动力、城镇困难人员、退役军人的就业工作。落实就业援助政策。加强就业技能培训。完善就业服务体系。

　　不断完善社会保障体系。积极推进五大社会保险扩大覆盖面。整合城乡基本养老保险和基本医疗保险制度。推进城镇职工基本医疗保险市级统筹，健全大医保信息系统；努力推进城乡居民医疗保险统筹。继续推进保障性安居工程建设，抓好质量安全和分配管理。抓好城乡低保和农村五保供养工作，加强城乡居民临时生活救助、救灾救济、医疗救助和法律援助，抓好残疾人社会保障和服务体系建设，妥善安排好困难群众的生产生活。抓好住房公积金制度扩面和规范缴存工作。

　　加快发展教育事业。全面实施素质教育，深入开展师德师风和行风建设，提高教育质量。切实支持湖南城市学院、益阳医专、湖南工艺美术职业学院、益阳职业技术学院建设，更好地发挥高校服务地方经济社会发展的作用。积极发展中等职业教育和幼儿教育。加大教育投入，大力改善办学条件，抓好合格学校建设，加快解决中心城区义务教育学位问题，进一步提高义务教育水平。完善优质教育资源共享机制，推进城乡教育均衡发展。

　　推动文化事业繁荣发展。构建富有特色的文博体系，完成“益阳印象”数字博物馆框架，启动市图书馆、群艺馆、国家级文物保护单位羊舞岭窑遗址公园和明清古街的规划建设。加强基层公共文化服务体系建设，完善乡镇（街道）、社区文化场所设备配置和管理服务，加强农家书屋建设及后续管理。扎实推进非物质文化遗产和历史文物保护工作。加快广播电视事业发展，抓好报刊网络、新闻出版、文艺创作、社会科学等工作。大力开展群众文化活动和文化演艺惠民活动。加快湖南工艺美术创意产业园建设，推动文化产业集聚发展。

　　努力提高人民健康水平。进一步提高城乡居民健康建档率。加强乡镇卫生院、社区卫生服务中心、村卫生室建设。提高参合农民医疗费用报销比例。开工建设市中心医院东部新区分院。提升疾病预防控制、妇幼卫生、卫生应急和采供血工作水平。继续抓好群众性应急救护培训。巩固完善基本药物制度。高度重视抓好食品安全工作，加强药品监督管理，确保人民群众饮食用药安全。

　　积极发展其他各项社会事业。抓好人口和计划生育工作，稳定低生育水平。深入推进全民健身运动开展。重视抓好气象、水文基础项目建设，加强探测观测环境保护，提升防灾减灾能力和服务水平。支持工会、妇联、共青团依照章程开展工作。积极推动义工（志愿者）事业发展。发展慈善事业、社会养老事业。加强全民国防教育，抓好国防动员和后备力量建设，支持武警、消防部队建设。认真做好第三次经济普查工作。切实抓好工商行政管理、质量技术监督、民族、宗教、外事、侨务、对台、人民防空、档案、保密、地方志、老龄、关心下一代等其他各项工作。

　　（九）强力推进社会管理创新，促进社会和谐稳定

　　坚持依法治市，广泛开展全民法制教育，提高全市人民的法律意识。加强社会主义核心价值体系学习教育，组织开展全市第三届道德模范评选表彰活动，推进各项群众性精神文明创建活动持续深入开展。全面落实安全生产责任制，积极推进安全生产标准化和隐患排查治理及应急救援体系建设，继续深入开展“打非治违”行动和重点行业领域安全专项整治，严防各类安全事故发生。大力整顿和规范市场经济秩序。加强市场物价调控监管，全面推进价格诚信体系建设，确保群众基本生活稳定。加强以“天网工程”为重点的社会治安防控体系建设，全面开展社区矫正工作，巩固“平安益阳”创建成果，建立健全防、管、控、打一体化的社会治安长效机制。加强国家安全教育，提高全社会安全防范意识。加大社会管理创新力度，深入推进社会稳定风险评估，开展突出不稳定因素大排查、大化解、大整治工作。完善突发公共事件应急处置机制，提高应急管理能力。切实做好群众工作，完善依法、理性、有序的信访工作机制，强化人民调解工作，深入推动“三调联动”建设，规范法律服务，维护社会大局稳定，努力实现公众安全感和群众满意率稳步提升。

　　（十）强力推进政府自身建设，努力打造人民满意政府

　　坚持为民办事。强化调查研究，畅通社情民意的收集渠道，把民生民利摆在第一位，认真解决群众关注的难点、热点问题，扎扎实实为群众办实事、办好事。坚持规范办事。自觉接受人大及其常委会的法律监督、工作监督，充分发挥政协在协商民主建设中的重要作用；坚持依法决策、科学决策、民主决策，深化政务公开，提高政务服务水平；推行行政问责、绩效管理、服务承诺、限时办结等制度，提升政府执行力。坚持快捷办事。进一步解放思想，开拓创新，切实改进行政管理，减少行政审批，大力推行网上政务服务，提高行政效能。改进文风会风，精简文件简报，没有实质内容，可发可不发的文件简报一律不发；开短会，开管用的会，说短话，力戒空话、套话。坚持干净办事。强化对政府公务人员的理想信念和廉洁自律教育，落实廉政建设责任制，规范行政权力运行，加强行政监察和审计监督，严肃查处各类违法违纪案件，创造风清气正的政务环境。

　　各位代表，益阳正处在后发赶超的关键时期，新的形势、新的任务催人奋进。让我们在中共益阳市委的坚强领导下，全面贯彻党的十八大精神，解放思想，坚定信心，迎难而上，扎实工作，努力让益阳人民生活得更加幸福、更有尊严！
